# Fructose vs. glucose: modulating stem cell growth and function through sugar supplementation

**DOI:** 10.1002/2211-5463.13846

**Published:** 2024-06-25

**Authors:** Salaheldeen Elsaid, Xiangdong Wu, Sui Seng Tee

**Affiliations:** ^1^ Department of Diagnostic Radiology and Nuclear Medicine University of Maryland School of Medicine Baltimore MD USA

**Keywords:** fructose, metabolism, stem cells

## Abstract

In multicellular organisms, stem cells are impacted by microenvironmental resources such as nutrient availability and oxygen tension for their survival, growth, and differentiation. However, the accessibility of these resources in the pericellular environment greatly varies from organ to organ. This divergence in resource availability leads to variations in the potency and differentiation potential of stem cells. This study aimed to explore the distinct effects of glucose and fructose, as well as different oxygen tensions, on the growth dynamics, cytokine production, and differentiation of stem cells. We showed that replacing glucose with fructose subjected stem cells to stress, resulting in increased *Hif1α* expression and stability, which in turn led to a reduction in cell proliferation, and alterations in cytokine production. However, fructose failed to induce differentiation of human mesenchymal stem cells (hMSCs) as well as mouse fibroblasts into mature adipocytes compared to glucose, despite the upregulation of key markers of adipogenesis, including C/EBPβ, and PPARγ. Conversely, we showed that fructose induced undifferentiated mouse fibroblasts to release cytokines associated with senescence, including IL1α1, IL6, IL8, MCP1, and TNF1α, suggesting that these cells were undergoing lipolysis. Taken together, our results suggest that altering the culture conditions through changes in hexose levels and oxygen tension places considerable stress on stem cells. Additional research is required to further characterize the mechanisms governing stem cell response to their microenvironments.

## Graphical abstract

Stem cells rely on nutrients and oxygen, which vary by organ, affecting their growth and differentiation. This study explored how sugar, and oxygen levels, impact stem cells. Fructose stresses stem cells, reducing their growth and changing cytokine production. Moreover, fructose didn't promote fat cell formation like glucose did. These findings highlight the importance of extracellular conditions for stem cell health.

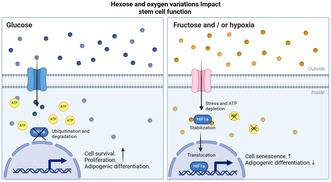

**Abbreviations**
C/EBPβ: CCAAT‐enhancer‐binding proteins beta
HIF1α: hypoxia inducing factor1 alpha
hMSCs: human mesenchymal stem cells
IL: interleukins
KHK: ketohexokinase
MCP1: monocyte chemoattractant protein
PPARγ: peroxisome proliferator‐activated receptor gamma
TNF1α: tumor necrosis factor1 alpha

The availability of sugars and oxygen in the stem cell microenvironment plays a pivotal role in their metabolism, proliferation, and differentiation [1, 2]. Notably, these resources are not infinite, but their usage is tightly regulated via cytokines and hormones, that orchestrate a network of differential metabolic fates [3, 4]. This includes the activation of membrane transporters and anabolic enzymes, facilitating the uptake of sugars and its conversion into cellular biomass [5, 6]. Hence, sugars and oxygen are essential components of cellular metabolism and are crucial for stem cell growth, differentiation, and cytokine expression.

At the same time, abnormally high levels of glucose and fructose in diabetic patients create a substantial stress to stem cells. This can lead to adverse effects on different stem cells by prompting their differentiation into new blood capillaries (angiogenesis) in the retina [5, 6, 7, 8] or adipocytes in bone marrow (adipogenesis) [1]. Therefore, the maintenance of hexoses at physiological levels supports regular metabolic and cellular functions, while its disruption induces a significant stress on stem cells.

Many studies have focused on differentiating stem cells into particular cell types, such as adipocytes [9], chondrocytes [1, 10], hepatocytes [2], and osteoblasts [11]. Other studies focused on their advantageous immunomodulatory properties as they secrete intrinsic cytokines that have the potential to mitigate inflammations and attenuate fibrosis [12, 13]. Consequently, stem cells are considered indispensable due to their unique combination of multipotency and immunomodulatory capabilities.

Stem cells can be isolated from various tissues that differ not only in their origin but also in their microenvironment. This microenvironment may differ in hexose concentrations and oxygen level [2, 10, 14]. Consequently, we aim to study the differential effect of different concentrations of glucose and fructose and distinct oxygen tensions on stem cells growth dynamics, cytokine production and differentiation.

## Materials and methods

### hMSC culture and differentiation

hMSCs were procured commercially, derived from a 20‐year‐old female, and were of bone marrow origin (Rooster Bio, RoosterVial hBM‐1M, Frederick, MD, USA). hMSCs were cultured in Iscove's modified Dulbecco's medium (IMDM) supplemented with 10% fetal calf serum (FCS). The culture medium also contained 100 IU·mL^−1^ of penicillin, 100 mg·mL^−1^ of streptomycin (P–S), and 10 ng·mL^−1^ of platelet‐derived growth factor BB (PDGF‐BB). Cells at 80–90% confluency were incubated for 3 weeks in adipogenic differentiation media which consisted of IMDM supplemented with 10% rabbit serum, 0.5 mm 3‐isobutyl‐1‐methylxanthin (IBMX), 1 μm hydrocortisone, 0.1 mm indomethacin, and P–S, in the absence of insulin. Media were changed every 3 days. MSCs were fixed with cold 10% formalin for 15 min, washed twice with PBS, and cytoplasmic triglyceride droplets were stained with BODIPY (Sigma, Rockville, MD, USA) for 15 min at room temperature (RT). Cells were washed and mounted with Vectashield containing DAPI nuclear stain. Cells were observed under Leica fluorescent microscope (Leica DMi8 system, Deerfield, IL, USA). All differentiation experiments were performed in cells passaged < 5 passages, to ensure robustness of hMSCs.

### Animal husbandry

All mice were housed in a specific pathogen‐free animal facility at 22 °C with a 12 h light–dark cycle with lights on from 7:00 to 19:00. During experiments, mice had free access to food and water. Post‐natal day 1 (P1) mice were utilized to isolate brown preadipocytes and euthanized with an overdose of Isoflurane inhalation, a procedure approved by the Institutional Animal Care and Use Committees (IACUC) at the University of Maryland, Baltimore (Protocol #0818019), consistent with recommendations by the American Veterinary Medical Association (AVMA) Guidelines for the Euthanasia of Animals.

### Isolation and immortalization of stromal vascular cells

Brown preadipocyte isolation and immortalization was previously described with modification [15, 16, 17]. Briefly, brown fat tissue was removed from interscapular region, minced into small piece, and digested in buffer including 1 mg·mL^−1^ collagenase II at 37 °C for 30–40 min digested tissues were then filtered through a 100 μm cell strainer into a new 50 mL sterile tube. Cells were then pelleted by centrifuge at 600 ***g*** for 5 min, and plated into a 6‐well culture plate with DMEM/F12, 10% FBS, and 1% penicillin–streptomycin and cultured in 37 °C and 5% CO_2_ for overnight. Cells were washed with pre‐warmed 1× PBS, then infected with retrovirus to express SV40 Large T antigen. The retroviral particles were packaged by 293T cells transfected with pVSV‐G, pCL‐Eco, and pBabe‐neolarge TcDNA plasmids. After Infecting for 48 h, cells were cultured in selection medium including with 450 μg·mL^−1^ neomycin. When reaching 80–90% confluence, cells were sub‐cultured to a new 6‐well plate. Cells were remained in neomycin selection for at least 7 days. All experiments were performed on cells < 10 passages.

### Differentiation of mouse primary brown adipocyte

Immortalized preadipocytes were grown in the DMEM medium supplemented with 1 g·L^−1^ glucose, 10% FBS, and 1% penicillin–streptomycin. When cells reached to 100% confluence, differentiation (day 0) was initiated with the induction medium with 1 g·L^−1^ glucose or 1 g·L^−1^ fructose supplemented with 0.5 mm IBMX, 20 nm insulin, 125 nm indomethacin, 1 nm T3, 1 μm dexamethasone, 1 μm rosiglitazone for 48 h. Cells were then incubated in the maintenance medium with 1 g·L^−1^ glucose or 1 g·L^−1^ fructose supplemented with 20 nm insulin, 1 nm T3 for 12 days.

Live cell lipid droplet staining was achieved by HCS LipidTOX deep red neutral lipid stain and imaged with fluorescence microscopy (Leica DMi8 system).

Differentiated mouse adipocytes were cultured in Dulbecco's modified Eagle's medium (25 mmol·L^−1^ glucose), supplemented with 10% (vol/vol) FBS, 1% penicillin, and streptomycin, and 1% Gultamax. Medium was exchanged every 3 days, and cells were trypsinized and reseeded at 1 : 4 dilution when 80–90% confluence was reached. All experiments were performed on cells < 10 passages.

### Cytokines measurement in the conditioned medium

Conditioned media were collected from either human MSCs or from mouse fibroblasts (preadipocytes). Samples were assayed for cytokines by the University of Maryland, Baltimore Cytokine Core Lab according to manufacturer's directions.

Briefly, a 96‐well plate (Greiner, Radnor, PA, USA) was wet with 200 μL of Assay Buffer and placed on a shaker for 10 min. The plate is then decanted and 25 μL of Assay Buffer or appropriate buffer is added to each well and 25 μL of standard/sample/control was added to the appropriate wells. Samples were run in duplicate for all samples. Then, 25 μL of a mixture containing required cytokines (1 : 50 dilution) that have been conjugated to beads is added. All plates contain at minimum high and low control in order to determine the validity of the plates. The plate is then placed on a shaker, at 4 °C overnight. The plate was then placed on a magnetic washer, 200 μL of Wash Buffer added to each well, the plate was set on a shaker at 60 g for 1 min and repeated an additional two times. After the last decanting step 25 μL of detection antibody is added and the plate is placed on a shaker for 1 h at room temp. Then, 25 μL of Phycoerythrin (1 : 25 dilution) is added to each well and the plate is placed back on the shaker for 30 min. The plate is then washed three times and 150 μL of Sheath Fluid is added to each well. The plate is then read using a Luminex MagPix reader. The data is then calculated using luminex's exponent Software (Rockville, MD, USA).

### RNA extraction and qPCR

Total RNA was extracted from 2 × 10^6^ HepG2 or Huh7 cells using PureLink RNA Mini Kit (Invitrogen, Waltham, MA USA) according to manufacturer instructions. iScript™ cDNA Synthesis Kit (Biorad, Hercules, CA, USA) was used to convert one microgram of high purity RNA into cDNA in a total reaction volume of 20 μL. The rection volume was diluted to 200 μL to reach (5 ng·μL^−1^) 0.2 μL was used for qPCR (10 ng) per reaction.

Two microliter (~10 ng of cDNA) of this cDNA was used for subsequent PCR amplification with SYBR™ Select Master Mix (cat. 4472908, Invitrogen) in quantstudio 5 (ThermoFisher Scientific, Waltham, MA, USA) using the following specific primers for human, Table 1 or for mice, Table 2 were purchased from Integrated DNA Technology, IDT (Coralville, IA, USA).

**Table 1:** Key resources.

Reagent or resource	Source	Identifier
Chemicals, peptides, and recombinant proteins		
PureLink RNA Mini Kit	Invitrogen	12183018A
iScript™ cDNA Synthesis Kit	Biorad	1708840
SYBR™ Select Master Mix	Invitrogen	4472908
MicroAmp Optical 96‐Well	Invitrogen	N8010560
SurePAGE, Bis‐Tris, 4–12%,10 wells	Genscript	M00653
Collagenase II	Sigma	C2‐28‐100MG
DMEM (no glucose)	Gibco	11‐966‐025
FBS	Fisher Scientific	SH3010903
Penicillin–streptomycin solution	Fisher Scientific	MT30001CI
Neomycin	Gibco	10131‐035
siRNA delivery medium	Accell	DRM‐B‐005000‐100
PhosSTOP	Roche	4906845001
IBMX	Sigma	410957
Insulin	Sigma	I5500
Indomethacin	Sigma	405268
Triiodothyronine (T3)	Sigma	T6397
Dexamethasone	Sigma	265005
Rosiglitazone	Sigma	PHR2932
LipidTOX deep red neutral lipid stain	Fisher Scientific	H34477
Superblock buffer	Invitrogen	37535
Antibodies		
Anti KHK	Sigma	HPA007040
Anti‐HIF1α (D2U3T)	Cell signaling	14 179
Anti‐PPARγ (C26H12)	Cell signaling	2443
C/EBP α	Cell signaling	8178
Anti‐α tubulin	Cell signaling	3873
Anti β‐Actin (Rb)	Cell signaling	2250
Plasmids and siRNA		
pVSV‐G	Addgene	138479
pCL‐Eco	Addgene	12371
pBabe‐neoLarge TcDNA	Addgene	1780
Human HIF1A 3091 siRNA	Accell	DRM‐A‐004018230005
Non‐targeting siRNA	Accell	DRM‐D‐0019100105
Experimental models: Organisms/strain, Cell lines		
Human MSC	Rooster Bio.	N/A
Fibroblasts isolated from interscapular brown fat pads of male C57BL/6 mice (WT)		N/A
Software		
Prism 9.0	GraphPad	https://www.graphpad.com/

**Table 2:** qPCR primers for *Homo sapiens*.

Human (*Homo sapiens*) primers			
Gene name	Forward	Reverse	Source
*Khk*	CTGCGTTGTGCAGACTCTATT	TCAGCGGGAGGAGGTATTT	IDT
*Igfbp3*	CGCTACAAAGTTGACTACGAGTC	GTCTTCCATTTCTCTACGGCAGG	IDT
*Igf1*	CTCTTCAGTTCGTGTGTGGAGAC	CAGCCTCCTTAGATCACAGCTC	IDT
*Hif1α*	TATGAGCCAGAAGAACTTTTAGGC	CACCTCTTTTGGCAAGCATCCTG	IDT
*Stra13*	TAAAGCGGAGCGAGGACAGCAA	GATGTTCGGGTAGGAGATCCTTC	IDT
*C/EBP β*	AGAAGACCGTGGACAAGCACAG	CTCCAGGACCTTGTGCTGCGT	IDT
*Pparγ*	TGCAGGTGATCAAGAAGACG	AGTGCAACTGGAAGAAGGGA	IDT
*Cpt1*	CCTCCGTAGCTGACTCGGTA	GGAGTGACCGTGAACTGAAA	IDT
*Atgl*	CCCACTTCAACTCCAAGGACGA	GCAGGTTGTCTGAAATGCCACC	IDT
*Hsl*	AGCCTTCTGGAACATCACCGAG	TCGGCAGTCAGTGGCATCTCAA	IDT
*Cd36*	CAGGTCAACCTATTGGTCAAGCC	GCCTTCTCATCACCAATGGTCC	IDT
*Tgf1 β*	TACCTGAACCCGTGTTGCTCTC	GTTGCTGAGGTATCGCCAGGAA	IDT
*IL1 β1*	CCACAGACCTTCCAGGAGAATG	GTGCAGTTCAGTGATCGTACAGG	IDT
*IL6*	AGACAGCCACTCACCTCTTCAG	TTCTGCCAGTGCCTCTTTGCTG	IDT
*β‐Actin*	CACCATTGGCAATGAGCGGTTC	AGGTCTTTGCGGATGTCCACGT	IDT

### Western blot analysis

Cells were lysed in RIPA buffer, 150 mm NaCl, 50 mm HEPES, pH 7.6, containing 0.1% of (100× Protease inhibitor) and one tablet of PhosSTOP (No. 4906845001). Equal amounts of protein samples were blocked in 4× loading buffer and run in 4–12% SurePAGE™, Bis‐Tris gel (No. M00653) then transferred to a PVDF membrane. After blocking with superblock buffer (INV‐37535), Immunoprobing the membrane with specific antibodies designated in Tables 1, 2, 3.

**Table 3:** qPCR primers for *Mus musculus*.

Mouse (*Mus musculus*) primers			
Gene name	Forward	Reverse	Source
*IL6*	TGGACCTTCCAGGATGAGGACA	GTTCATCTCGGAGCCTGTAGTG	IDT
*Cpt1*	GGTGCCTATGTCTCAGCCTCTT	GCCATAGAACTGATGAGAGGGAG	IDT
*Cd36*	GGACATTGAGATTCTTTTCCTCTG	GCAAAGGCATTGGCTGGAAGAAC	IDT
*Tgf1ß*	TGATACGCCTGAGTGGCTGTCT	CACAAGAGCAGTGAGCGCTGAA	IDT
*Mcp1*	GCTACAAGAGGATCACCAGCAG	GTCTGGACCCATTCCTTCTTGG	IDT
*Igfbp3*	CCTCAATGTGCTGAGTCCCAGA	CTTGTCCACACACCAGCAGAAG	IDT
*Atgl*	GGAACCAAAGGACCTGATGACC	ACATCAGGCAGCCACTCCAACA	IDT
*Hsl*	CAGACCTCAAGTGGAACCAGCA	CGGAGAATGTTCCTCAGTAGTGG	IDT
*Hif1α*	CCTGCACTGAATCAAGAGGTTGC	CCATCAGAAGGACTTGCTGGCT	IDT
*β‐Actin*	CATTGCTGACAGGATGCAGAAGG	TGCTGGAAGGTGGACAGTGAGG	IDT

### Statistical analysis

Data were analyzed with graphpad prism 9 software (GraphPad Software, Boston, MA, USA) and presented as Mean ± SEM. We used one‐way ANOVA followed by *post hoc* Tukey test for multiple comparison. Statistical significance is indicated as figures using the following denotations, *P < 0.05, **P < 0.01, ***P < 0.001, ****P < 0.0001.

## Results

### Fructose dampens human bone marrow‐derived stem cell proliferation

Standard IMEM growth medium consists of 25 mm glucose. We investigated cellular growth under increasing concentrations of either glucose or fructose as the sole carbon source in human bone marrow‐derived mesenchymal stem cells (hMSC). Of note, we selected concentrations of glucose that mimic physiological concentrations (5 mm), as well as two other concentrations reported to mirror prediabetic and diabetic conditions at 10 and 25 mm, respectively [18]. Fructose is present at much lower concentrations in circulation, but the bone marrow has been reported to contain > 5 mm fructose [19], providing the justification for testing the ability of hMSCs to grow at 5‐, 10‐ and 25 mm fructose.

**Fig. 1:**
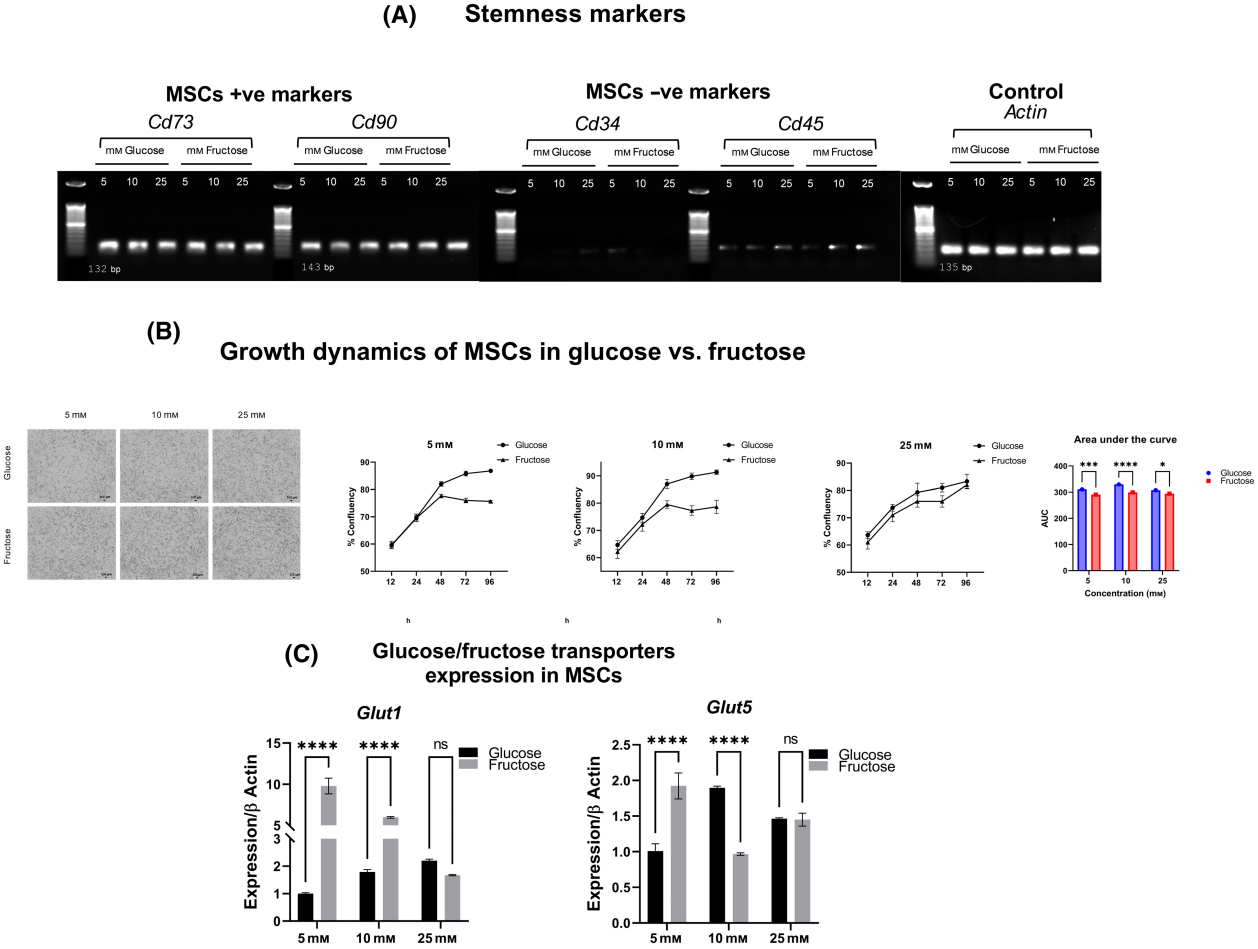
Fructose preserves stemness but decreases cell proliferation hMSC. (A) Agarose gel electrophoresis of quantitative PCR (qPCR) products with primers from either positive or negative markers of stemness, with housekeeping gene, Actin. To indicate molecular size, a 50 bp ladder was used. (B) Representative images of MSC grown in either glucose or fructose as the sole carbon source, and growth curves of MSCs at concentrations of 5, 10, and 25 mm of either hexose, quantified by the area under the growth curves after 96 h. (C) GLUT expression in hMSC, grown in 5, 10, and 25 mm of either hexose, with expression of each transporter quantified relative to the housekeeping gene, Actin. Data represented as mean ± SEM (*n* = 3). All *P* values were calculated using one‐way ANOVA followed by *post hoc* Tukey test for multiple comparison with *P < 0.05, **P < 0.01, ***P < 0.001, and **** *P* < 0.0001.

At all concentrations of sugar tested, we report that hMSC retained stemness markers (positive markers CD73 and CD90, and negative markers CD34 and CD45) [20], suggesting that this defect in proliferation was not connected to the loss of pluripotency (Fig. 1A). However, growth dynamics were dampened when hMSCs were grown in 5‐, 10‐ and 25 mm fructose compared to glucose at equivalent concentrations. This is summarized in Fig. 1B.

In addition, we quantified glucose transporter expression as a potential modulator of hMSC growth in different hexoses (Fig. 1C). Two main types of transporters are responsible for glucose uptake, the sodium‐glucose linked transporters (SGLT) and facilitated diffusion glucose transporters (GLUT) [21]. Here, we report the absence of SGLT transcripts in hMSC (Fig. S1). In contrast, GLUT1, a ubiquitous transporter with broad tissue expression [22], showed a hexose concentration‐dependent increase in expression in both glucose and fructose. While we failed to detect GLUT2 and ‐4 (Fig. S1), GLUT5, previously described as a fructose transporter in enterocytes, only demonstrated a fructose‐induced increase in expression relative to glucose, at 5 mm hexose. Taken together, while studies in cancer cells have described hexose transport as a limiting factor in fructose‐specific growth [23], hMSC robustly upregulate GLUTs, but is insufficient to rescue reduced proliferation in these pluripotent cells.

### Fructose alters hMSC secretory phenotype

The striking differences in growth dynamics between fructose and glucose prompted us to investigate cytokine production as a possible mediator of differential cell growth. Mammalian cells require both nutrients, and growth factor signaling to stimulate proliferation [24]. Thus, we proceeded to quantify the secretory phenotypes of hMSC, grown in different hexoses.

**Fig. 2:**
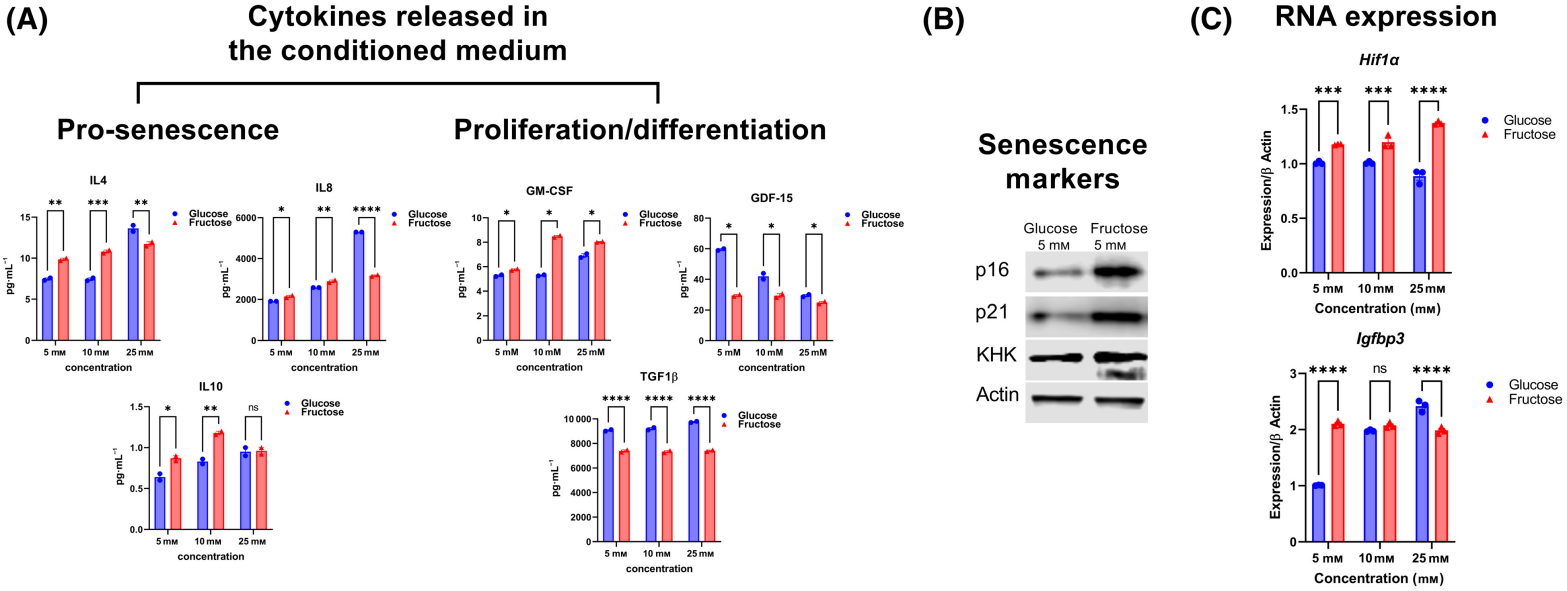
Fructose modulates cytokine production. (A) Fructose increased pro‐senescent cytokines, reduced pro‐proliferative factors, and increased differentiation‐associated factors. (B) Western blot analysis of senescence marker, p16 and p21 as well as the fructolytic enzyme ketohexokinase (KHK). (C) MSC grown in fructose showed an elevation of Hif1α transcript compared to equivalent glucose‐grown cells, as well as expression of Igfbp3, a downstream Hif1α target. Data represented as mean ± SEM (*n* = 3). All *P* values were calculated using one‐way ANOVA followed by *post hoc* Tukey test for multiple comparison with *P < 0.05, **P < 0.01, ***P < 0.001, and **** P < 0.0001.

Interestingly, hMSCs under fructose significantly release more senescence‐associated cytokines, such as interleukins (IL)‐4, ‐8, and ‐10 [25, 26, 27, 28]. Beyond senescence, an increase in granulocyte macrophage colony stimulating factor (GM‐CSF) was observed, that is classically associated with reduced stem cell proliferation, instead favoring differentiation into monocyte lineage [29]. Conversely, tumor growth factor (TGF)1β and growth/differentiation factor (GDF)‐15, both pro‐proliferative cytokines [30] was reduced, suggesting fructose‐grown MSC as functionally distinct from glucose‐grown counterparts (Fig. 2A).

Noting the change in senescence‐associated cytokines, we asked if fructose modulated expression of intracellular cell senescence markers, specifically, p16 and p21. p16INK4a or p16, is an inhibitor of cyclin‐dependent kinases and plays a critical role in cell cycle regulation, enforcing the G1 phase cell cycle arrest in senescent cells [31, 32, 33]. Similarly, p21CIP1/WAF1 or p21 prevents cell cycle progression [33, 34]. Western blot analysis of MSCs cultured in 5 mm fructose showed a significant increase in immunosignal of both markers (Fig. 2B). Thus, fructose not only enhances the production of anti‐proliferative cytokines but also activates cell senescence markers in hMSCs.

Next, we investigated possible mechanisms that promoted an anti‐proliferative phenotype in fructose‐grown hMSC. We focused on hypoxia induced factor (HIF)‐1α, as this transcription factor has previously been shown to cause senescence in high hexose microenvironments, ultimately leading to cellular senescence [35, 36]. Notably, one of the significant challenges in stem cell transplantation is cell death induced by Hif1α activation [37].

We found that *Hif1α* was upregulated in the fructose‐treated cells. Additionally, we investigated transcript levels of insulin‐like growth factor binding protein (*Igfbp)‐3* as a direct indicator of *Hif1α* [38, 39] transcriptional activity. Consistently, *Igfbp3* mRNA levels were upregulated at 5 and 25 mm fructose (Fig. 2C). Taken together, fructose promoted differential cytokine production when grown in fructose, associated with senescence and an anti‐proliferative phenotype.

### Hypoxia alters hMSC function via upregulation of HIF1α

We showed that substituting glucose with fructose impacts proliferation with a concomitant elevated expression of *Hif1α* and *Igfbp3* under normoxic conditions. This suggested that fructose‐exposed MSC may experience metabolic stress, similar to that present in low oxygen tensions mediated by *Hif1α*. Notably, stem cells can be found in tissues that receive low levels of oxygen such as in the bone marrow [1, 40] and within expanding white adipose tissue [41, 42]. Thus, we hypothesized that MSC grown in hypoxia will phenocopy MSC grown in fructose as the sole carbon source.

**Fig. 3:**
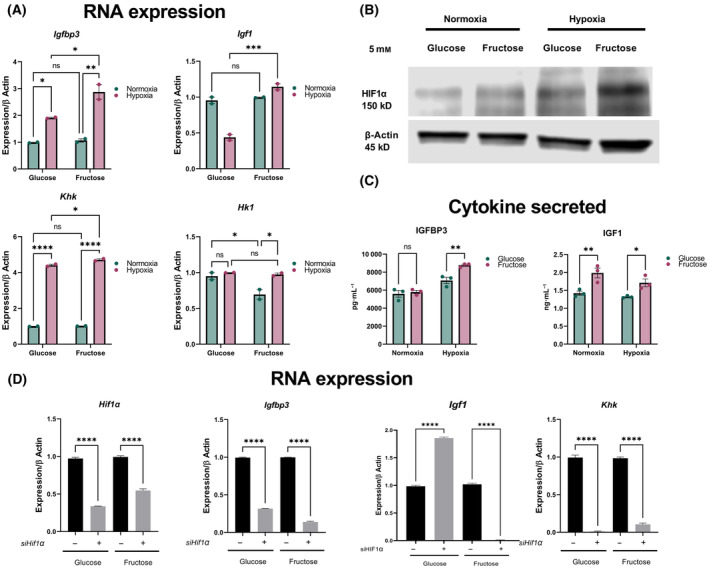
Hypoxia alters MSC function, that is further exacerbated by the presence of fructose. (A) MSC grown in 5 mm hexose exposed to either normoxia or hypoxia demonstrated changes in genes associated with cytokine production and fructose metabolism. (B) Hypoxia stabilizes Hif1α, that is maximally induced in fructose. (C) Cytokine release of IGFBP3 and IGF1 was maximally observed under hypoxia and fructose. (D) Abrogation of Hif1α expression under normoxic conditions reversed upregulation of genes associated with cytokine production and fructose metabolism, suggesting a central role for this transcription factor mediating altered MSC function in the presence of fructose. Data represented as mean ± SEM (*n* = 3). All *P* values were calculated using one‐way ANOVA followed by *post hoc* Tukey test for multiple comparison with *P < 0.05, **P < 0.01, ***P < 0.001 and **** P < 0.0001.

We previously observed elevated *Igfbp3* in MSC grown in 5 mm fructose compared to glucose. A similar phenotype was observed when MSC were grown in hypoxia (1% O_2_) compared to normoxia (21% O_2_), where *Igfbp3* transcripts were also elevated under low oxygen tensions. Ketohexokinase (KHK) levels were also increased in hypoxia, suggesting the ability of low oxygen to promote fructolysis, phenocopying elevated KHK seen under high‐fructose conditions. In contrast, hexokinase (HK)‐1 transcripts were not significantly changed (Fig. 3A). Reassuringly, Hif1α stabilization was observed under hypoxia, regardless of hexose source. Interestingly, fructose appears to promote the highest retention of Hif1α levels (Fig. 3B). Furthermore, IGFBP3 and IGF1 released by MSCs in the culture medium closely matched the mRNA expression levels. (Fig. 3C). Together, these results suggest the ability of low oxygen tensions to alter MSC cytokine production and fructose metabolism, potentially via Hif1α‐dependent mechanisms.

To demonstrate a central role for Hif1α, we proceeded to abrogate this transcription factor using small interfering RNA (siRNA). HIF1 transcripts were reduced 72‐h after knockdown, confirming reduced transcription factor abundance. Accordingly, *Igfbp3* transcripts were also reduced post‐knockdown, suggesting the ability of hypoxia to alter the MSC secretory phenotype is partly mediated by Hif1α. Strikingly, KHK transcripts were significantly downregulated upon abrogation of HIF1α, suggesting that fructose metabolism in MSC may be intimately linked to oxygen tension (Fig. 3D). Taken together, hypoxic conditions recreate many of the phenotypes seen when MSC are grown in fructose, that can be reversed when HIF1α transcriptional activity is abolished.

### Fructose dampens hMSC differentiation to adipogenic fate

Beyond defects in proliferation and cytokine secretion, we next explored the impact of fructose on stem cell differentiation. hMSC are multipotent cells that can be differentiated into several distinct cell types, including adipocytes [9] and chondrocytes [1, 10]. Here, we started by investigating the effect of substituting glucose with fructose as the sole carbon source in differentiation medium at various hexose concentrations (5, 10, and 25 mm) in hMSC.

**Fig. 4:**
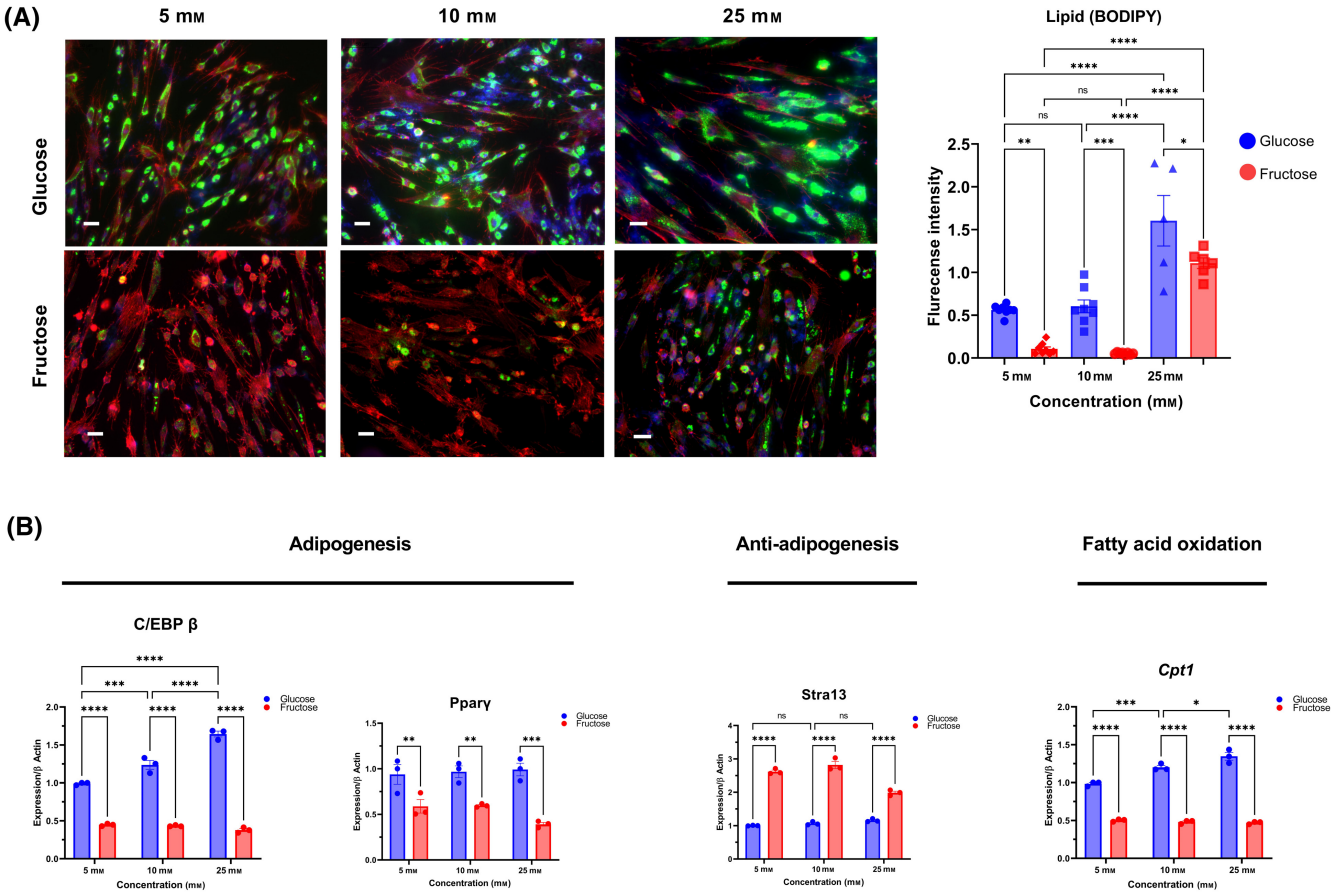
Fructose modulated Adipogenesis in MSCs Compared to Glucose. (A) MSCs were cultured in adipogenic differentiation medium supplemented with either glucose or fructose. After 14 days of differentiation, cells were fixed with 4% PFA, and lipid accumulation was visualized using BODIPY stain (green), F‐Actin (red), and nuclei (blue). (A) Lipid accumulation was observed across a spectrum of glucose concentrations, ranging from 5 to 25 mm. In contrast, the induction of adipogenic differentiation was only achieved at the 25 mm fructose concentration (B). Fructose‐treated cells showed elevated levels of IL1β1 and a reduction in Cpt1 expression. Scale bar = 100 μm. Data represented as mean ± SEM (*n* = 3). All *P* values were calculated using one‐way ANOVA followed by *post hoc* Tukey test for multiple comparison with *P < 0.05, **P < 0.01, ***P < 0.001 and **** P < 0.0001.

In glucose, significant hMSC differentiation was observed, visualized as accumulation of lipid droplets by BODIPY staining. Conversely, when hMSCs were exposed to fructose, adipocyte differentiation and the subsequent accumulation of lipids displayed a noticeable reduction (Fig. 4A). Of note, total cell number remained unchanged, suggesting a fructose‐induced block in differentiation, not cell viability.

Mechanistically, hMSCs exposed to fructose showed a significant decrease in the expression levels of adipogenic markers, specifically CCAAT/enhancer‐binding protein [43] (*C/EBPβ*) and peroxisome proliferator‐activated receptor [44] (*Pparγ*). In contrast, *Stra13*, a transcription factor often associated with the repression of adipogenesis [15] was upregulated. Intriguingly, fructose also increased the expression of carnitine palmitoyltransferase (Cpt1) and interleukin (IL)‐1β1, indicating a reduced capacity for fatty acid oxidation. (Fig. 4B and Fig. S2). Taken together, fructose is associated with a failure to induce master adipogenic transcription factors, while favoring lipolytic gene expression.

### Fructose dampens differentiation potential of preadipocytes

While fructose clearly impacted hMSC growth, function, and differentiation, we proceeded to ask if these defects were hMSC‐specific, or if these phenotypes extend to stem cells from different origins. Thus, we proceeded to use primary preadipocytes, isolated from intrascapular, brown adipose tissue (BAT) depots of neonatal mice [16, 17, 45], which are also able to assume an adipogenic fate.

**Fig. 5:**
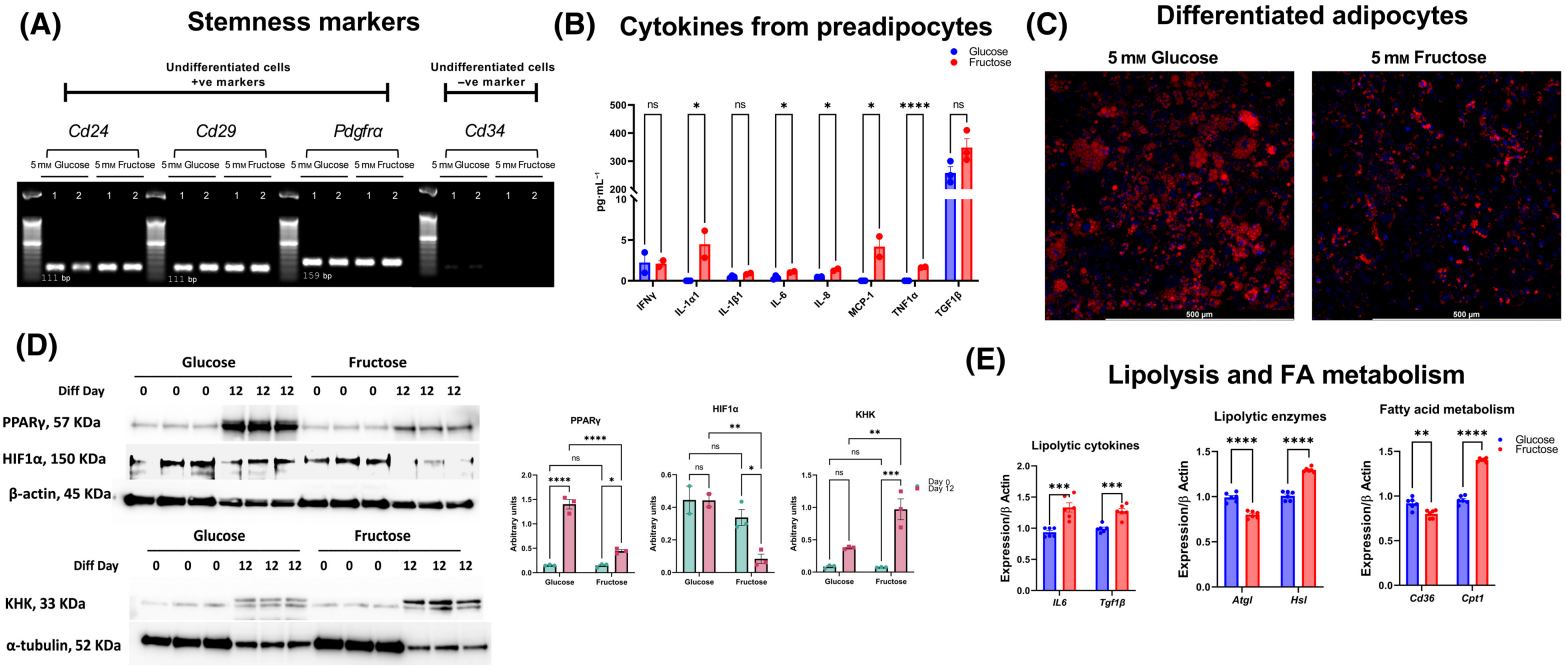
Fructose hindered adipogenesis in murine primary BAT preadipocytes. (A) Preadipocytes retained markers of stemness in both glucose and fructose. To indicate molecular size, a 50 bp ladder was used. (B) Preadipocytes showed elevation of senescence‐associated cytokines when exposed to fructose (C) Preadipocytes were differentiated in adipogenic differentiation medium supplemented with either 5 mm glucose or fructose, for 12 days and lipid accumulation was visualized using neutral red stain red, and nuclei, blue. (D) Western blot showing PPARγ, a major adipogenic factor at day 0 and at day 12, and HIF1α. Both proteins were downregulated in the fructose‐treated cells. (E) An Increase in Hsl, a lipolytic enzyme was associated with upregulation in IL6 and TGF1β expression, concomitant with Cpt1 upregulation, a maker for FA oxidation. Data represented as mean ± SEM (*n* = 3). All *P* values were calculated using one‐way ANOVA followed by *post hoc* Tukey test for multiple comparison with *P < 0.05, **P < 0.01, ***P < 0.001 and **** P < 0.0001.

First, we confirmed that murine BAT preadipocytes maintained expression of stemness‐associated markers, in all hexose concentrations tested, as seen with hMSC (Fig. 5A). While pluripotency of BAT preadipocytes remain unaltered, these preadipocytes secreted higher levels of senescence‐associated cytokines when cultured in fructose, specifically IL1α1, IL6, IL8, monocyte chemoattractant protein (MCP1), and tumor necrosis factor (TNF1 α) (Fig. 5B).

Beyond impacting preadipocyte function, we proceeded to examine the role of fructose in murine BAT preadipocyte differentiation. Here, we report that fructose dampened preadipocyte differentiation, seen as a remarkable reduction in lipid accumulation fructose‐treated cells (Fig. 5C). This reduction in lipid accumulation coincided with a decrease in PPARγ, a key transcription factor for adipogenesis, but with an increase in KHK, suggesting the ability of preadipocytes to upregulate fructolytic machinery (Fig. 5D).

Interestingly, fructose‐grown preadipocytes upregulated IL6 and Tgf1β, both recognized for their capacity to stimulate lipolysis [46, 47, 48]. Consistently, transcripts of the lipolytic enzyme, hormone‐sensitive lipase (Hsl) was also upregulated. At the same time, expression Cpt1 and CD36, both involved in fatty acid oxidation, were also elevated (Fig. 5E). In sum, fructose failed to support differentiation of murine preadipocytes, but instead demonstrated an elevation of genes associated with catabolic fatty acid metabolism.

## Discussion

Stem cells play a vital role in tissue regeneration and wound healing [49, 50]. They greatly vary not only in their origins but also in their potency [51]. This can be attributed partially to the special microenvironment surrounding them that contains the required nutrient, oxygen level, and cytokines suitable for survival and differentiation [51, 52]. Any change in the pericellular environment will consequently modulate cell signals, cytokines, that will act directly on the cell itself (autocrine effect) or neighboring cells (paracrine effect) [51, 52, 53]. Therefore, preserving the pericellular environment of stem cells is crucial for their normal function.

For instance, elevated glucose and fructose levels in diabetic patients exert significant stress on stem cells within various organs [54, 55]. High fructose can generate toxic metabolites such as uric acid that in turn elevates ROS production [56], and elevated advanced glycation end‐products (AGE), that have been associated with insulin resistance and dyslipidemia [57]. Notably, fructose is significantly more reactive than glucose to form AGE via the Maillard reaction, due to the keto group present in fructose [58]. Beyond the formation of toxic by‐products, fructose metabolism leads to ATP depletion that triggers apoptosis in primary hepatocytes [59, 60]. This stress leads to increased expression of Hif1α, a factor known for promoting adverse effects in multiple organs, including angiogenesis in retina [61] and white adipose tissue [62], and osteoporosis [1, 40]. HIF‐1α is subject to rapid ubiquitination under normal oxygen conditions [63]. However, in hypoxic environments or when exposed to prolyl hydroxylases (PHDs) inhibitors such as ROS, HIF‐1α becomes more stable and translocate into the nucleus [63]. Hence, elevated hexose and ROS production can induce stress leading to HIF1a stabilization and modulation of stem cells viability.

In this study, we report the novel observation that replacing glucose with fructose also subjects stem cells to stress, resulting in increased Hif1a expression and stability. This led to a reduction in cell proliferation and alterations in cytokine production. The increase of Hif1a is evident through its induction of Igfbp3 expression and secretion [38]. Notably, Igfbp3 triggers apoptotic pathway and inhibits cellular proliferation [39, 64]. Conversely, we found that Igfbp3 expression decreased when Hif1a was knocked down, regardless of the sugar type used. The induction of Hif1a influence the production of cytokines associated with the senescence‐related phenotype [37, 65], aligning with our findings that demonstrate an increase in P16 and P21 immunosignals. Interestingly, we showed that IL4, IL8, and IL10 were increased in the fructose containing medium along with the reduction in GDF15, a growth factor that supports proliferation [30]. These three interleukins are associated with senescence phenotype [25, 28]. Hence, fructose exerts stress on stem cells associated with increased Hif1a and activation of senescence‐associated phenotype.

The previous findings prompted us to ask a question whether this phenomenon extends to differentiation. To address this, we conducted a comparative study of the adipogenic differentiation potential of human MSCs, which are multipotent, and mouse fibroblasts isolated from the interscapular brown adipose tissue. These mouse fibroblasts are more specialized and can only differentiate into brown adipocytes [66]. This contrast in their differentiation capabilities offers an excellent model for examining how the replacement of glucose with fructose might influence their differentiation.

Interestingly, fructose failed to induce differentiation of human MSCs as well as mouse fibroblasts into mature adipocytes compared to glucose. Key markers of adipogenesis, including C/EBPβ, and PPARγ [41] were upregulated in fructose‐treated MSCs but did not reach the threshold required for adipogenesis when compared to glucose (Fig. S1A). Conversely, we showed that fructose induces undifferentiated mouse fibroblasts to release cytokines associated with senescence, including IL1α1, IL6, IL8, MCP1, and TNF1α. This observation prompted us to investigate further by differentiating these cells into adipocytes. Intriguingly, in fructose‐treated mouse fibroblasts, there was a remarkable increase in IL6, a well‐known cytokine that stimulates lipolysis [46, 47, 48]. This was supported by the significant upregulation of *Hsl* concomitant with the increase in *Cpt1* expression. These changes suggest that these cells were undergoing lipolysis, with fatty acids being oxidized to generate energy.

Surprisingly, hMSCs exhibited elevated IL1β1 and IL6 expression also known for inducing lipolysis [66, 67]. Notably, the expression of lipolytic enzymes Atgl and Hsl remained unchanged while Cpt1 was downregulated. Hence, fructose impedes adipogenic differentiation by elevating the expression of cytokines that promote both lipolysis and the oxidation of fatty acids. Notably, insulin signaling has previously been implicated as a driver of differential fatty acid metabolism in adipocytes [68]. In our experiments, hMSC were differentiated in the absence of insulin, suggesting that fructose‐induced upregulation of lipolysis in these cells as potentially insulin‐independent, consistent with published work, where fructose metabolism is generally seen as independent of this growth factor [69].

The wide‐ranging effects of fructose of stem cell growth and function adds complexity to the multi‐faceted consequence of diets high in added sugars such as fructose. While the impact of fructose has been best described in non‐alcoholic fatty liver disease (NAFLD), our study clearly shows that fructose alters the physiology of both differentiated and undifferentiated cells. It is notable that fructose impacts hMSC growth negatively, while hepatocyte proliferation has previously reported to be the same, in either glucose or fructose as the sole carbon source [70]. Furthermore, while stem cells appear to upregulate lipolysis in the presence of fructose, induction of lipogenesis is a classical hallmark of fructose in hepatocytes [71]. Together, these data highlight the importance of considering the pleiotropic consequences of sugar metabolism, in cells and tissues of different origins.

In conclusion, our study has revealed that altering the culture conditions through changes in hexose levels and oxygen tension places considerable stress on stem cells. This stress not only leads to shifts in cytokine composition but also activates pathways associated with senescence or inhibits the maturation of stem cells into adipocytes. This study opens the door to further investigations into the mechanisms governing stem cell response to their microenvironments.

## Conflict of interest

The authors declare no conflict of interest.

### Peer review

The peer review history for this article is available at https://www.webofscience.com/api/gateway/wos/peer‐review/10.1002/2211‐5463.13846.

## Author contributions

SE planned, performed experiments and curated data, as well as prepared and edited manuscript. XW performed experiments and curated data. SST prepared and edited manuscript, supervised and finalized data.

## Supporting information


**Fig. S1.** Expression of stemness marker and hexose transporters in hMSCs.
**Fig. S2.** Fructose modulates adipogenesis, inflammatory markers, and fatty acid metabolism in hMSCs.

## Data Availability

All data needed to evaluate the conclusions in the paper are present in the paper and/or the Supplementary materials.
