# Supplementary material for: Fructose vs. glucose: modulating stem cell growth and function through sugar supplementation
Source: FEBS Open Bio. 2024 Jun 25;14(8):1277–90. doi: 10.1002/2211-5463.13846 (PMC11301265; doi:10.1002/2211-5463.13846)
Supplement: Supplementary file 1 — Fig. S1. Expression of stemness marker and hexose transporters in hMSCs. Fig. S2. Fructose modulates adipogenesis, inflammatory markers, and fatty acid metabolism in hMSCs. [file FEB4-14-1277-s001.docx]

**Supplementary Figures**

| 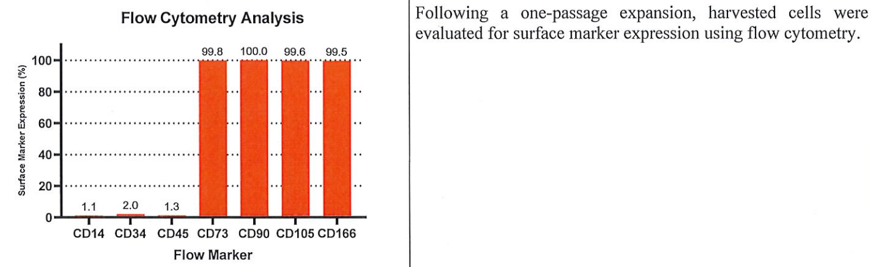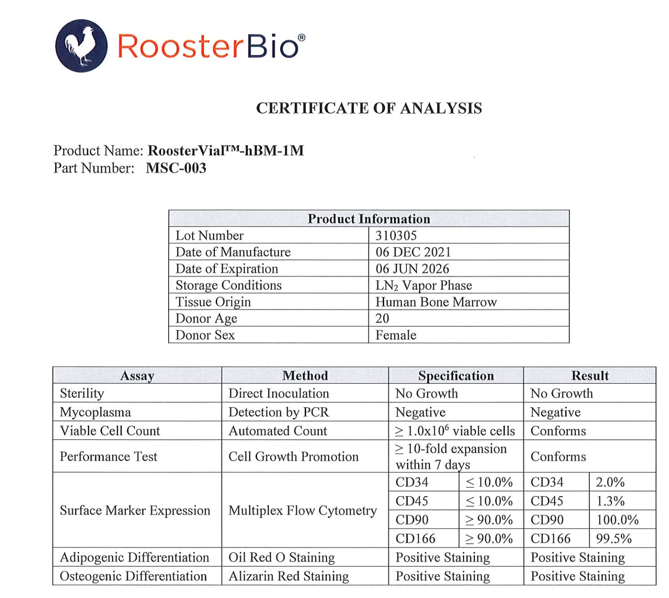 |
| --- |
| 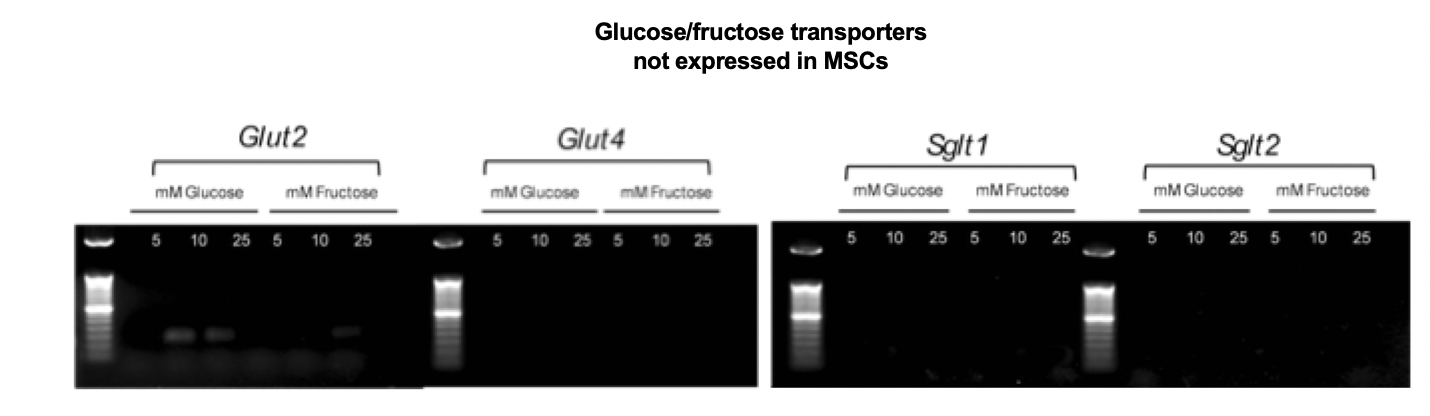 |
|  |

**Supplemental Figure 1: Expression of stemness marker and hexose transporters in hMSCs:** hMSC retain stemness markers, but do not express GLUTS 1 and 4, as well as SGLT1 and SGLT2. To indicate molecular size, a 50 bp ladder was used.

|  |
| --- |
|  |
|  |

**Supplemental Figure 2: Fructose modulates adipogenesis, inflammatory markers, and fatty acid metabolism in hMSCs.** (A) MSCs under fructose showed significant upregulation of C/EBPβ and Pparγ but not enough to induce adipogenesis. (B) Proinflammatory cytokines IL1β1 and IL6 are known for their ability to induce lipolysis as well as Cd36, a fatty acid transporter, showed significant upregulation in MSCs differentiated in fructose. (C) Conversely, Hsl, a lipase that is directly activated via interleukin as well as Atgl, remained unchanged. Data represented as mean ±SEM (n=3). All *P* values were calculated using one-way ANOVA followed by Post HOC Tukey test for multiple comparison with **P* < 0.05, ***P* < 0.01, and ****P* < 0.001.
